# Osteoporosis in the setting of knee arthroplasty: a narrative review

**DOI:** 10.1186/s42836-024-00273-z

**Published:** 2024-10-02

**Authors:** Mohammad Daher, Elio Mekhael, Mouhanad M. El-Othmani

**Affiliations:** 1 Orthopedic Department, Hôtel Dieu de France, Beirut, Lebanon; 2 Department of Orthopedic Surgery, Brown University Medical Center, Providence, RI 02906 USA

**Keywords:** Osteoporosis, Knee arthroplasty, Osteoarthritis, Knee replacement, Bone mineral density

## Abstract

Patients undergoing knee replacement, which is mainly indicated in severe osteoarthritis, are frequently co-affected by osteoporosis and osteopenia. With a prevalence standing at around 20% in patients receiving knee arthroplasty, osteoporosis could lead to poor outcomes postoperatively. Some of these complications include periprosthetic fractures and an increased revision rate. Antiresorptive medications have been shown to be beneficial postoperatively. However, no studies have been conducted on whether they had any benefits if given preoperatively. Surgical management may also be beneficial, but this area remains full of controversy.

## Introduction

Osteoporosis is a relatively common degenerative bone disorder with an estimated prevalence of 18.3% across the globe [1]. Within the United States, 10 million Americans older than 50 years suffer from osteoporosis, with an estimated additional 34 million at risk of developing the disease [2]. The disease process affects bone quantity and constitutes a spectrum that is characterized by a progressive decrease in bone mineral density (BMD). This imbalance results in an increased risk of fragility fractures, which constitute a substantial morbidity and mortality risk among the frail geriatric population [3, 4].

Osteoarthritis is a frequently concomitant disease affecting geriatric patients. The worldwide prevalence of osteoarthritis has increased by 113.25% between 1990 and 2019, with the knee joint being cited as the most affected site [5–7]. As a result, the annual rate of total knee arthroplasty (TKA) is continuously increasing in the world and the US, with 4.7 million Americans receiving TKA in 2010 [8].

In patients undergoing primary TKA, osteoporosis can pose substantial technical challenges and has been noted to be associated with a higher risk of postoperative complications and revisions [9]. As such, the prevalence and severity of osteoporosis carry considerable implications for patients undergoing TKA. Thus, the purpose of this manuscript was to provide a critical appraisal of the literature and explore the prevalence of osteoporosis and osteopenia among patients undergoing TKA, describe postoperative outcomes, and highlight the available evidence on potential perioperative medical and surgical interventions to optimize outcomes and reduce any potential associated risks.

## Data collection

PubMed was searched until December 2023 for the qualified papers. Using Boolean Operators, a combination of the keywords “knee arthroplasty” OR “knee replacement” AND “osteoporosis” OR “Osteopenia” OR “Bone mineral density” OR “BMD” was used. Reference lists from papers and online searches were also used to find literature.

## Incidence and screening

With an aging population and continually increasing functional demand, the number of performed and projected TKA continues to grow, and the number of patients with osteopenia or osteoporosis undergoing the procedure is expected to follow suit. Studies have shown that a large number of TKA candidates were diagnosed with osteoporosis (17.4%) and osteopenia (45.9%) [10] with prevalence reaching 60% in postmenopausal women awaiting TKA [11]. In a recent study, Ishi et al. reported that among TKA recipients, the prevalence of dual-energy X-ray absorptiometry (DXA) defined osteoporosis was 22% in women and 5% in men [12]. Moreover, in a recent meta-analysis, Xiao et al. noted that 64% of total joint arthroplasty patients suffered from osteopenia and osteoporosis [13]. Interestingly, the authors reported a notably lower treatment rate at 32.9% in those with a confirmed diagnosis[13]. Similarly, Ha et al. noted a prevalence of osteoporosis of 50% among patients scheduled for TKA, of which only 15.1% had pharmacological treatment prior to the procedure [14]. The percentage of patients with osteoporosis receiving treatment prior to arthroplasty has been reported as low as 5% in other studies [15].

While several screening tools for identifying osteoporosis and osteopenia have been described, determining which patients benefit from presurgical DXA remains challenging. As a simple screening tool, investigating the history of “disease-defining” fractures, such as distal radius fractures, vertebral compression fractures, and hip fractures could be incorporated into the assessment of TKA candidates, and could alert clinicians for underlying and undiagnosed osteoporosis. Dual-energy computed tomography (DECT) could provide an alternative and has been shown to be an accurate estimator of BMD, whose findings correlates with the results provided by central DXA [16]. The importance of preoperative DXA in the identification of orthopedic surgical patients at risk of osteoporosis has been proven in several joints such as the hip, shoulder, spine, and specifically the knee. The authors of a retrospective cohort study proposed a simple screening protocol defining who should obtain DXA due to high osteoporosis risk which included: females ≥ 65, males ≥ 70, fracture history when ≥ 50 years, or FRAX major osteoporotic fracture risk (without bone mineral density [BMD]-adjustments) ≥ 8.4%. For patients receiving DXA, screening sensitivity levels reached 96% for identifying T-score osteoporosis (T-score ≤  −2.5 as defined by the WHO) and 99% for identifying modified clinical osteoporosis (T-score ≤  −2.5, BMD-adjusted FRAX risk or prior hip/spine fracture) with only 1 osteoporosis patient not meeting screening criteria recommending DXA [17]. This being said, DXA remains the gold standard for the diagnosis of osteoporosis, with a T score ≤  −2.5 defining osteoporosis, and a T score between −1 and −2.5 defining osteopenia [17]. More recently, CT scans of the knee with machine learning models have reportedly been capable of detecting up to 91.2% of the cases [18].

## Impact on outcomes

Numerous studies evaluated the impact of reduced BMD on the perioperative outcomes following TKA. In a recent study, Chang et al. noted significantly higher rates of complications and revisions following TKA among patients with osteoporosis, even after controlling for patient demographics [19]. Osteoporosis or osteopenia predisposes a patient to a higher risk of perioperative complications such as fracture, implant migration, and aseptic loosening [15]. Kang et al. [20] reported that reduced bone density correlated with the risk of intraoperative distal femur fractures. The authors noted a BMD cutoff value of −2.8 significantly increased the risk for intraoperative fractures by 2.3 folds (*P* = 0.042). However, one must note that, in their surgeries, the authors did put the femoral component first in their TKA which they showed to be a risk factor for intraoperative distal femur fractures [20]. Furthermore, Harris et al. noted that osteoporosis was associated with a 2-fold increased risk of 5-year revision for periprosthetic fractures after TKA [9]. In addition, Holzer et al. [21] exhibited a significant association between the fracture risk assessment tool (FRAX) and the incidence of suffering from periprosthetic fractures in both total hip and knee arthroplasty. Even in navigation-assisted TKA, osteoporosis was found to affect tibial component position (*P* = 0.039), rendering osteoporotic knees in more valgus [22]. This was explained by the higher likelihood of undetected intraoperative pin motion in these affected knees, which subsequently impacts the accuracy of planned bone cuts and component positioning [22].

Other studies evaluated the effect of prior fragility fractures on the perioperative outcomes of TKA. Fragility fractures can be the initial clinical manifestation in a lot of patients. Agarwal et al. [23] reported an increase in risk by 2-fold (*P* < 0.001) for periprosthetic fractures and 3-fold (*P* < 0.001) for future fragility fractures 8 years postoperatively in patients with a fragility fracture within 3 years prior to TKA. In addition to these risks, and at 2-year follow-up, Albright et al. [24] noted an increased incidence of hospital readmissions (*P* < 0.001), non-infection-related revision surgery (*P* = 0.002), prosthesis dislocations (*P* < 0.001), and deep periprosthetic infections (*P* < 0.001) in patients with a fragility fracture within 3 years prior to TKA. Interestingly, the authors noted a lack of statistical significance of the increased revision rate (3.0% in the control group vs. 3.1% in the group with a prior fragility fracture; *P* = 0.215), which was attributed to appropriate surgeon decision-making [23].

Nevertheless, other studies reported the absence of a negative impact of osteoporosis on the outcomes after TKA. Huang et al. [25] showed that lower bone density was associated with reduced postoperative pain and improved subjective ratings of functional outcomes. However, the study included 43 patients with a follow-up duration of six months, constituting a small cohort with a short duration that might be not long enough to accurately predict long-term results [25]. Similarly, Watanabe et al. evaluated the prognosis of TKA patients and found that knee function ratings and the severity of osteoporosis did not correlate with outcomes, concluding that the latter does not adversely affect TKA results [26]. On the other hand, other studies highlighted the increased risk of tibial component migration in cementless TKA in patients with lower BMD [27, 28]. However, in the latter, local BMD was found to be affected by local osteoarthritis [29] and was less reflective of the patient’s general bone health status when compared to systemic BMD [30, 31].

## Management

### Medical management

The majority of studies assessing the medical management of osteoporosis and osteopenia analyzed the effect of postoperative drugs and supplements on local periprosthetic bone health instead of whole-body BMD. Vitamin D supplementation is among the most widely studied interventions in the setting of low BMD conditions. Barker et al. [32] administrated a multivitamin containing 900 IU of vitamin D postoperatively to patients undergoing TKA and noted a significantly decreased postoperative inflammatory response. However, this finding might be limited by the effect of other concomitantly administered vitamins. Furthermore, another study compared 800 to 2000 IU of vitamin D postoperatively and found no difference in the rate of postoperative falls or the Western Ontario and McMaster Universities Osteoarthritis Index (WOMAC) [33]. Nevertheless, as the authors did not include a control group with a placebo instead of a lower dose of vitamin D, the effectiveness of the latter cannot be studied (Table 1).

**Table 1:** Studies exploring the use of medical therapy in osteoporotic patients undergoing total knee arthroplasty (BMD = Bone Mineral Density)

Drug	Author	Year	Results
Vitamin D	Barker et al.	2021	Multivitamin supplement containing 900 IU of vitamin D significantly decreased postoperative inflammatory response.
	Bischoff-Ferrari et al.	2018	No difference was found in the rate of postoperative falls or WOMAC index when comparing postoperative 800 to 2000 IU of vitamin D.
Bisphosphonate containing cement	Mastuszewska et al.	2022	Increased levels of osteoprotegerin and decreased RANKL and therefore decreased bone resorption
Alendronate	Wang et al.	2003	Reduced BMD loss at the distal femur and proximal tibia
	Soininvaara et al.	2002	Alendronate reduced early BMD loss at the metaphyseal anterior, posterior, diaphyseal, and metaphyseal regions of the femur.
	Wang et al.	2006	A six-month course of alendronate increased BMD at 6 (4.8%) and 12 (1.6%) months. No significant difference was noted after 36 months when compared to controls (−3.9% vs. −12.2%, respectively; *P* = 0.08).
	Jaroma et al.	2015	Lateral tibial metaphysis was increased with Alendronate until seven years (*P* = 0.002), and was significantly higher than that observed in the control group throughout (*P* = 0.024). No significant differences were found between the groups in the central femoral metaphyseal, tibial medial metaphyseal, or diaphyseal regions of either the femur or tibia.
	Teng et al.	2015	Long-term use of bisphosphonates was correlated with a significantly decreased risk of implant revision after total knee arthroplasty (TKA)
Denosumab	Murahashi et al.	2019	Improvement in BMD decreased at around 3 months postoperatively compared to 6 months with bisphosphonates and reduced tibial bone atrophy 12 months postoperatively
	Ledin et al.	2019	Denosumab reduced early migration of the tibial component.
Teriparatide	Suzuki et al.	2018	Increased periprosthetic BMD in the posterior and lateral regions of the condyles at 6 months and in the anterior and posterior regions of the condyles and the tibial diaphysis at 12 months
	Kaneko et al.	2016	Weekly injection of teriparatide after cementless TKA promoted bone ingrowth mostly in the medial aspect of the bone-prosthesis interface.

With regards to anti-osteoporotic drugs, a study assessing bisphosphonates-containing-cement in TKA showed an increase in the level of osteoprotegerin and a decreased receptor activator of nuclear factor kappaB ligand (RANKL), thus acting on important molecular elements to decrease bone resorption [34]. While these findings highlight interesting results at the cellular level, the clinical implications are yet to be determined. At a clinical level, the efficacy of a postoperative treatment with a specific bisphosphonate for a duration of 6 months, alendronate was shown to reduce the BMD loss at the distal femur and proximal tibia [35]. This postoperative improvement in BMD decline using alendronate was reported by multiple studies [36–39]. Interestingly, Murahashi et al. assessed the impact of postoperative treatment with denosumab, another anti-resorptive medication, on BMD following TKA. The authors noted an improvement in BMD decrease at around 3 months postoperatively compared to 6 months with bisphosphonates, and a reduced tibial bone atrophy 12 months postoperatively. [40]. Another study by Ledin et al. [41] assessing the impact of denosumab showed a reduced early migration of the tibial component. Nevertheless, one must be cautious when using this particular drug since its discontinuation without an alternative anti-resorptive drug can cause rebound-associated vertebral fractures, with a reported increased rate from 1.2 to 7.1 per 100 participant-years [40, 42–45]. Another anti-osteoporotic agent that can be added to the perioperative therapeutic arsenal is teriparatide, an anabolic agent. In fact, as compared to other osteoporosis drugs, this anabolic drug increases periprosthetic BMD instead of attenuating its decline, providing an effective and attractive intervention among this population [46, 47]. Furthermore, in a case–control study looking at risk factors for periprosthetic fractures, Park et al. showed that a history of osteoporosis medication reduced this risk [48]. No recommendations could be made yet as this is an area that needs to be much more intensively studied due to the lack of scientific articles assessing the medical management of osteoporosis in osteoporotic patients undergoing TKA.

### Surgical considerations

With regards to surgical management, intraoperative technique, and implant selection can affect perioperative osteopenia/osteoporosis and bone tissue remodeling. In a recent meta-analysis, Prince et al. reported that average BMD loss following TKA evolved as follows: 9.3%, 13.2%, 15.8%, and 15.4% at 3, 6, 12 and 24 months, respectively. The authors stress the rapid BMD decrease in the first 6 months after surgery and is then sustained to 24 months, highlighting the effect of bone health on the incidence of periprosthetic fracture [49].

The choice of implant fixation strategy in this population is critical and remains controversial. A study showed that, in the medial and anterior region below the tibial components, BMD was shown to be decreased for cemented components by 8.6% and 4.2%, respectively, until 24-month follow-up. On the other hand, cementless components were associated with an increased BMD of 1.8% and 7.4%, respectively, until the 24-month follow-up [50]. However, one must note that they excluded osteoporotic patients from their study. Moreover, a cemented mobile-bearing component was shown to prevent BMD loss after TKA whereas when the fixed-bearing component was adopted, BMD of the femur decreased. This difference was found to be statistically significant at 18 and 24 months (*P* < 0.05), proving that a cemented mobile-bearing component could have a beneficial effect on the BMD of the femur after TKA [51]. However, Ueyama et al. demonstrated, in their prospective cohort study, that there was no difference in peri-prosthetic BMD changes between mobile- and fixed-bearing prostheses in patients undergoing oral bisphosphonate therapy. This study suggested that the effect of oral bisphosphonate therapy might offset the influence of prosthetic design [52]. In addition, when comparing periprosthetic BMD in patients across all ages, osteoporotic and non-osteoporotic, managed with uncemented trabecular metal and cemented tibial components, Minoda et al. showed that the postoperative decrease in BMD in the lateral aspect of the tibia was significantly less in knees with trabecular metal components than in knees with cemented tibial components at twenty-four months (6.7% ± 22.9% vs. –36.8% ± 24.2%; *P* = 0.002) [53]. Petersen et al. found that hydroxyapatite coating of the tibial component did not exert any significant effect on the bone remodeling pattern of the proximal tibia [54]. The authors also showed that, in patients who underwent uncemented porous-coated TKA, BMD increased by 22% proximal to the fixation pegs [55]. Nevertheless, one must note that while cementless fixation was associated with a lower revision rate in men, it showed a higher rate of revision in women > 65 years of age[56]. Furthermore, in a national database study, Dubin et al. revealed that there was a higher risk of periprosthetic fractures at 5 years after TKA in osteoporotic patients undergoing cementless fixation when compared to cemented fixation [57] (Table 2).

**Table 2:** Studies exploring surgical measures in osteoporotic patients undergoing total knee arthroplasty (BMD = Bone Mineral Density)

	Author	Year	Results
Cemented vs. cementless components	Linde et al.	2022	BMD was decreased for cemented components by 8.6% and 4.2% in the medial and anterior regions, respectively, until 24-month follow-up. BMD was increased for cementless components by 1.8% and 7.4% in the medial and anterior regions, respectively, until 24-month follow-up.
	Dubin et al.	2024	The cementless cohort had a 5-year periprosthetic fracture risk following TKA of 7.8% (95% CI, 5.56 to 10.98) in comparison to 4.30% in the cemented cohort (85% CI, 3.98 to 4.65) (*P* < 0.0001).
	Ryan et al.	2024	Cementless fixation was associated with a lower revision rate in men, and it showed a higher rate of revision in women > 65 years of age
Mobile- vs. fixed-bearing components	Minoda et al.	2010	Prevention of BMD loss after TKA with mobile-bearing component compared to significant BMD loss of the femur with fixed-bearing component
	Ueyama et al.	2020	No difference was found in peri-prosthetic BMD changes between mobile- and fixed-bearing prostheses in patients undergoing oral bisphosphonate therapy. The BMD changes results are likely affected by oral bisphosphonate therapy.
Uncemented trabecular metal vs. cemented tibial components	Minoda et al.	2010	Less decrease in BMD in the lateral aspect of the tibia with trabecular metal components (6.7% ± 22.9% vs −36.8% ± 24.2%; *p* = 0.002).
Hydroxyapatite coating of tibial component	Petersen et al.	2005	No significant effect was found on bone remodeling pattern of the proximal tibia.
Uncemented porous-coated component	Petersen et al.	1995	Increased BMD by 22% proximal to the fixation pegs
PCL retention	Ishii et al.	2017	No substantial effect was found on the BMD of the proximal femur and tibia.
Medial peg position	Minoda et al.	2022	The closer the tibial medial peg was to the cortex, the larger the BMD loss in the medial part of the tibia at 2 years postoperatively.
Porous titanium construct Regenerex and porous coated (PPS) tibial tray	Winther et al.	2016	Both groups maintained peri-prosthetic BMD in lateral, medial and distal aspects below the central stem regions, with up to 8.1% and 6.5% increase at 12 months for the PPS and Regenerex, respectively.

As for the prosthesis design, Ishii et al. showed that, when measured approximately 10 years after TKA, PCL retention had no substantial effect on the BMD of the proximal femur and tibia [58]. Furthermore, Minoda et al. reported that BMD decreased in the medial region after the operation (*P* < 0.001), which was affected by the medial peg position. The closer the tibial medial peg was to the cortex the larger the BMD loss in the medial part of the tibia at 2 years postoperatively [59]. Moreover, a prospective study including 60 patients scheduled for TKA suggested that both the novel porous titanium construct Regenerex and the well-proven standard porous coated (PPS) tibial tray have a favorable effect in maintaining periprosthetic BMD in lateral, medial, and distal aspects below the central stem regions, with up to 8.1% and 6.5% increase at 12 months for the PPS and Regenerex, respectively [60].

## Conclusion

For some candidates undergoing TKA whose characteristics need to be further defined by future studies, preoperative screening may be advised to determine which patients can benefit from a perioperative intervention. To lessen the hazards related to individuals undergoing TKA with a low BMD, medical management of the latter could be implemented. Currently, the data show that cemented fixation is favored in osteoporotic patients to reduce postoperative periprosthetic fractures. Nevertheless, more studies addressing the effectiveness of preoperative treatment with anti-osteoporotic medication as well as intraoperative surgical modifications in preventing the adverse outcomes of TKA in osteoporotic patients are needed.

## Data Availability

Not available.
